# Penetrant-Triggered Responsiveness in Carboxylate Polymer of Intrinsic Microporosity

**DOI:** 10.1021/acsmacrolett.6c00475

**Published:** 2026-10-08

**Authors:** Maria S. Tavera-Villamizar, Durga Acharya, Cara M. Doherty, Anita J. Hill, Michele Galizia

**Affiliations:** † School of Sustainable Chemical, Biological and Materials Engineering, 6187 University of Oklahoma, 100E. Boyd Street, Norman, 73019, Oklahoma, United States; ‡ 2221 CSIRO Technology, Research Way, 3168, Clayton, Australia; § Materials Science and Engineering Program, The University of Oklahoma, 660 Parrington Oval, Norman, 73019, Oklahoma, United States

## Abstract

While complex mixture fractionation has become a primary focus in membrane science, the mechanisms behind penetrant-penetrant and penetrant-polymer interactions governing molecular separations require deeper investigation. Of equal importance, how the external environment dictates these interactions remains poorly understood. To address this knowledge gap, we elucidate the molecular mechanisms that control how microporous polymers structurally adapt to their external environment. Resolving previous speculations, we show that upon reaction with carboxylic acid groups in carboxylate polymer of intrinsic microporosity (cPIM), ammonia forms ammonium carboxylate adducts. This chemical modification alters small-molecule sorption, local chain organization, free volume distribution, and, ultimately, penetrant diffusion. Notably, ammonia-modified cPIM rapidly reacts with carbon dioxide, causing *in situ* polymer ionic cross-linking. Using a suite of advanced experimental methods, including transport measurements, FTIR, PALS, and NMR, we uncover the mechanisms behind the observed responsiveness and its time-evolution. Ultimately, we demonstrate that accounting for coupled interactions is crucial for accurate performance prediction and optimal design of responsive membranes.

## Graphical abstract

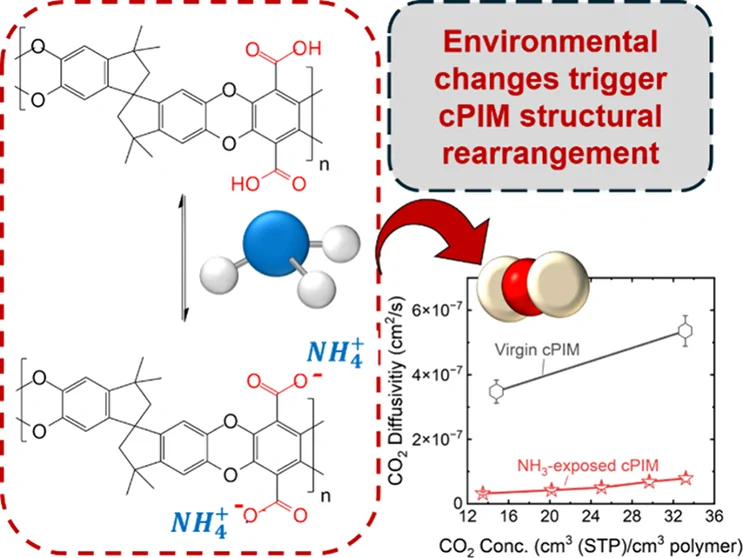

Complex mixture fractionation demands a fundamental shift in membrane science toward a deeper understanding of molecular interactions and their time evolution.^1–4^ Sorp-vection,^5^ as well as preferential pathways,^6^ are recently discovered transport mechanisms that leverage specific penetrant-penetrant and penetrant-polymer interactions to enable precise molecular separations.

Understanding how the external chemical environment affects these interactions is critical for designing adaptive materials, particularly in the context of modern microporous glassy polymers.^4,7–11^ Here, we investigate the stimuli-responsive behavior of carboxylate polymers of intrinsic microporosity (cPIM) to ammonia and carbon dioxide across multiple time scales. This study decodes the precise molecular mechanisms behind this responsiveness for the first time, resolving previous speculations.^12–14^ These fundamental insights provide a framework to improve molecular separations, such as NH_3_/CO_2_, and chemical sensors.

Details of PIM-1 synthesis and subsequent acid hydrolysis to cPIM were provided previously,^15^ and are summarized in section 1, Supporting Information.

Ammonia vapor sorption isotherm at 35 °C in a cPIM film exhibiting a degree of hydrolysis of 56% is shown in Figure 1. Details of the experimental procedure are provided in section 2, Supporting Information. After completing the first sorption run, a full vacuum was pulled overnight, after which sorption was measured again on the same sample (i.e., second run).

**Figure 1:**
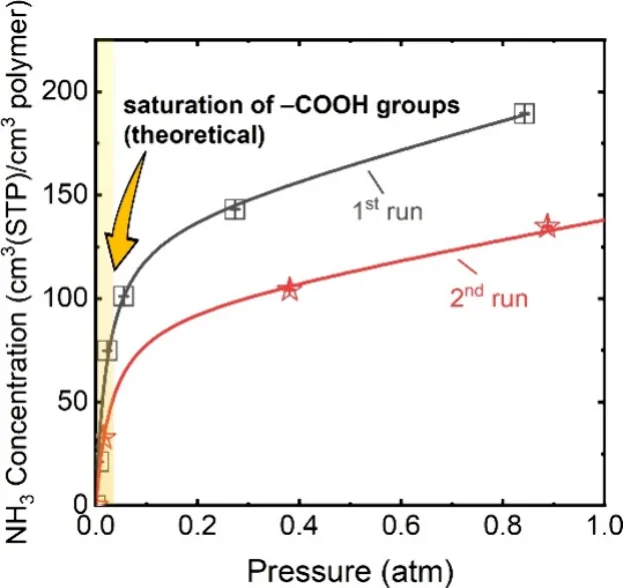
Ammonia sorption isotherm in cPIM at 35 °C (runs 1 and 2). Error bars were calculated using the error propagation method.^16^ Continuous lines represent dual mode fitting. The shaded yellow region represents the pressure at which theoretical full conversion of carboxylate groups in cPIM to ammonium carboxylate takes place during the first sorption run.

Ammonia sorption during the second run is 30% lower compared to virgin cPIM (i.e., first run). This result is unexpected, as second run sorption experiments with condensable penetrants generally show an increased uptake due to polymer swelling and conditioning.^17,18^ It is hypothesized that the formation of ammonium carboxylate during run 1, promoted by the reaction between ammonia and carboxylic acid groups in cPIM, depletes the available carboxylic acid sites, thereby inhibiting ammonia interaction in the second run. Stoichiometric calculations were performed to determine whether complete conversion of carboxyl groups to ammonium carboxylate is theoretically possible and, in case, at which pressure. During run 1, ammonia concentration in the polymer equates that of −COOH groups (i.e., ∼ 0.00276 (mol −COOH)/(cm^3^ polymer)) at 0.015 atm. Although purely theoretical, this calculation suggests that the decrease in ammonia sorption in the second run experiment is compatible with the saturation of carboxyl groups in cPIM.

Gas and vapor sorption in glassy polymers comes from two contributions,^19^ one associated with penetrant dissolution in transient gaps between polymer chains (i.e., Henry’s mode), and the other associated with penetrant adsorption into excess free volume (i.e., Langmuir’s mode):

1 $$C_{i}=k_{D,i}p+\frac{C_{H,i}^{’}b_{i}p}{1+b_{i}p}$$

where *C*_*i*_ is the penetrant concentration in the polymer, *p* is the pressure, *k*_*D,i*_ is the Henry’s constant, *C*_*H,i*_^′^ is the Langmuir’s sorption capacity, and *b*_*i*_ is the Langmuir’s affinity parameter. The Henry’s and Langmuir’s affinity constants estimated during run 1 exceed those estimated during run 2, indicating that ammonia molecules interact more favorably with the virgin sample than the pre-exposed sample (cf., Table 1). The decrease of the Langmuir capacity *C*_*H*_^′^ in the second run could be ascribed to partial occupation of excess free volume by bulky ammonium carboxylate groups. As discussed later, *Positron Annihilation Lifetime Spectroscopy* (PALS) data support this hypothesis.

**Table 1:** Dual-Mode Parametrization of Ammonia Sorption in cPIM at 35 °C^a^

	$k_{D}\left(\frac{cm^{3}\left(STP\right)}{cm^{3}polymeratm}\right)$	$C{’}_{H}\left(\frac{cm^{3}\left(STP\right)}{cm^{3}polymer}\right)$	*b* (*atm* ^–1^)
*1st ammonia sorption run*	64.6 ± 17.7	138.0 ± 12.8	43.4 ± 10.9
*2nd ammonia sorption run*	45.0 ± 2.1	95.86 ± 2.90	32.1 ± 3.7

a Uncertainty was calculated using the error propagation method.^16^

Similar to sorption, two subsequent ammonia permeation experiments were run at 35 °C (cf., Figure 2A). Experimental details are available in section 3, Supporting Information. First, ammonia permeability was measured in the virgin sample. Then, vacuum was pulled overnight, to closely mimic the sorption test, and a second ammonia permeability measurement was run on the same sample.

**Figure 2:**
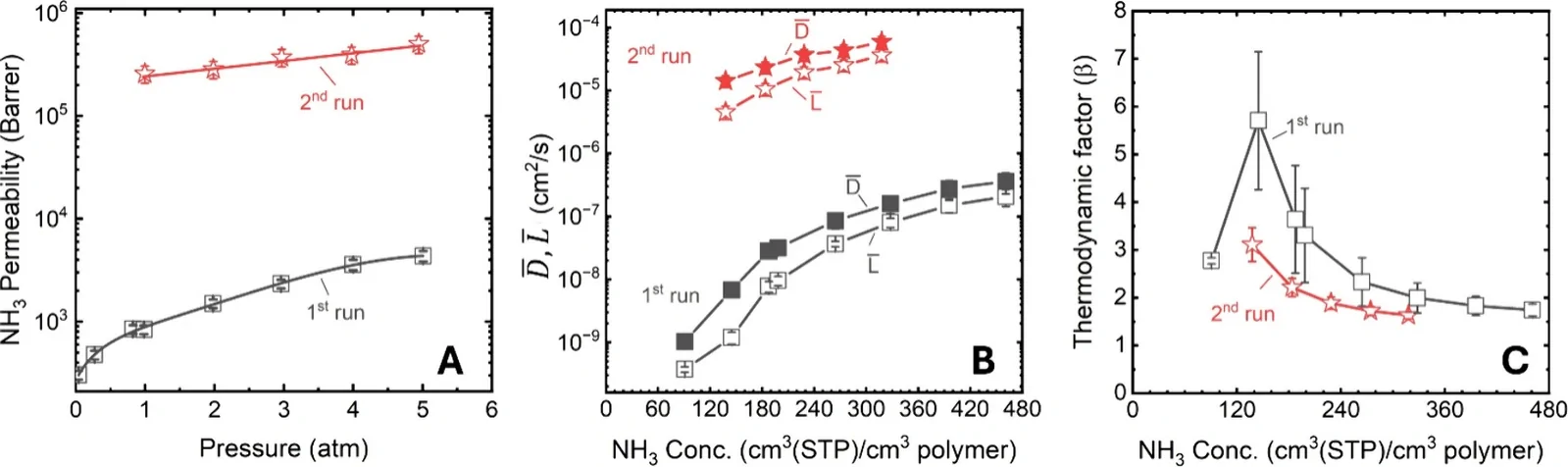
A) Ammonia permeability in cPIM at 35 °C as a function of pressure. B) Ammonia diffusion (*D̅*) and mobility (*L̅*) coefficients in cPIM at 35 °C. C) Thermodynamic factor (β) of ammonia in cPIM at 35 °C as a function of the ammonia concentration in the polymer. Results of the first and second run measurements are compared. Error bars were calculated using the error propagation method.^16^

Ammonia permeability in virgin cPIM (i.e., in the first run) increases monotonically with pressure. This behavior, indicative of polymer plasticization, is consistent with the large ammonia uptake in the polymer, owing to favorable interactions with the −COOH groups. If plasticization were to dominate the sorption process, however, an increase in ammonia uptake would be observed in the second run. This picture suggests that the reduced ammonia sorption during the second run is governed by enthalpic factors (i.e., inhibited penetrant-polymer interactions) rather than entropic factors, reflecting the conversion of −COOH groups to ammonium carboxylate.

Ammonia permeability during the second run exceeds by 2 orders of magnitude that measured during the first run. Consequently, the permeability behavior, controlled by plasticization, sharply contrasts with the sorption behavior, which is governed by polymer-penetrant interactions.

The ammonia diffusion coefficient in cPIM was calculated via the solution-diffusion model, using the experimental sorption and permeability coefficients.^15^ The sharp increase in diffusivity with pressure mirrors that in permeability, due to polymer plasticization (cf., Figure 2B).

The ammonia diffusivity in cPIM measured during the second run exceeds by 3 orders of magnitude that measured during the first run. Therefore, two separate behaviors emerge: ammonia equilibrium sorption decreases in the second run relative to the first run, due to suppressed polymer-penetrant interaction (i.e., enthalpic factors), while ammonia permeability and diffusivity increase, due to plasticization (i.e., entropic factors). The increased diffusivity and permeability in the second run are aligned with PALS data, which, as discussed later, show a significantly larger number of micropores in cPIM after exposure to ammonia.

To better understand the transport mechanism, the ammonia diffusion coefficient in cPIM, *D̅*_*i*_, was corrected for thermodynamic non-idealities, accounting for the interactions between NH_3_ and the −COOH groups. To do so, *D̅*_*i*_ was decomposed into its two contributions,^20,21^ the mobility factor *L̅*_*i*_ and the thermodynamic factor β. Specifically, if the chemical potential gradient across the membrane is assumed as the driving force for penetrant transport, *D̅*_*i*_ is the product of a kinetic parameter, named the mobility coefficient (i.e., thermodynamically corrected diffusion coefficient), *L̅*_*i*_, which accounts for the frictional resistance offered by the polymer to ammonia diffusion, and a thermodynamic factor, β, related to penetrant-polymer interactions. If β = 1, penetrant-polymer mixing is ideal, therefore *D̅*_*i*_ = *L̅*_*i*_. Otherwise, β can either be larger than 1 if interaction is attractive, or less than 1 if interaction is repulsive. *L̅*_*i*_ and β can be calculated from sorption and diffusion data as follows:^20,21^

2 $${\mathrm{L̅}}_{i}=\frac{{\mathrm{D̅}}_{i}}{\beta }={\mathrm{D̅}}_{i}\frac{\partial \ln\omega_{i}}{\partial \ln a_{i}}$$

where *ω*_*i*_ is the ammonia mass fraction in cPIM and *a*_*i*_ is the ammonia activity, calculated as the ratio between the ammonia pressure and its saturation pressure. As shown in Figures 2B–C, the ammonia mobility, *L̅*_*i*_, in the virgin cPIM membrane increases with increasing ammonia concentration, consistent with sorption-induced plasticization. The thermodynamic factor, β, is above 1, confirming attractive interactions with cPIM. In the virgin sample, β initially increases and then decreases with penetrant concentration. This behavior indicates that at low concentrations ammonia molecules strongly interact with the −COOH groups in cPIM. However, as the interacting sites get converted to ammonium carboxylate, additional ammonia uptake does not result in stronger interactions. Therefore, the thermodynamic factor starts decreasing with increasing concentration.

The diffusivity and mobility factors in the second run are higher than in the first run, confirming that ammonia plasticizes cPIM. Finally, the thermodynamic factor in the second run sample is lower relative to the first run. This result indicates that in the second run most carboxyl groups are converted into ammonium-carboxylates, making the ammonia-cPIM interaction less likely to occur.

To demonstrate that carboxyl groups react with ammonia to form ammonium carboxylate, as shown in Figure 3A, the FTIR signatures of cPIM before and after exposure to ammonia at 5 atm are compared in Figure 3B. Upon exposure to ammonia, the band at 1720 cm^–1^, associated with the cPIM carbonyl stretching vibration,^15^ appears severely attenuated, which is compatible with carbonyl groups experiencing complexation with ammonia. A new band appears at 1590 cm^–1^, which indicates deprotonation of carboxylic groups. Finally, the band centered at 1670 cm^–1^ is assigned to the *N - H* symmetric bending of the complex *COO* ^–^ *NH*_4_^+^.

**Figure 3:**
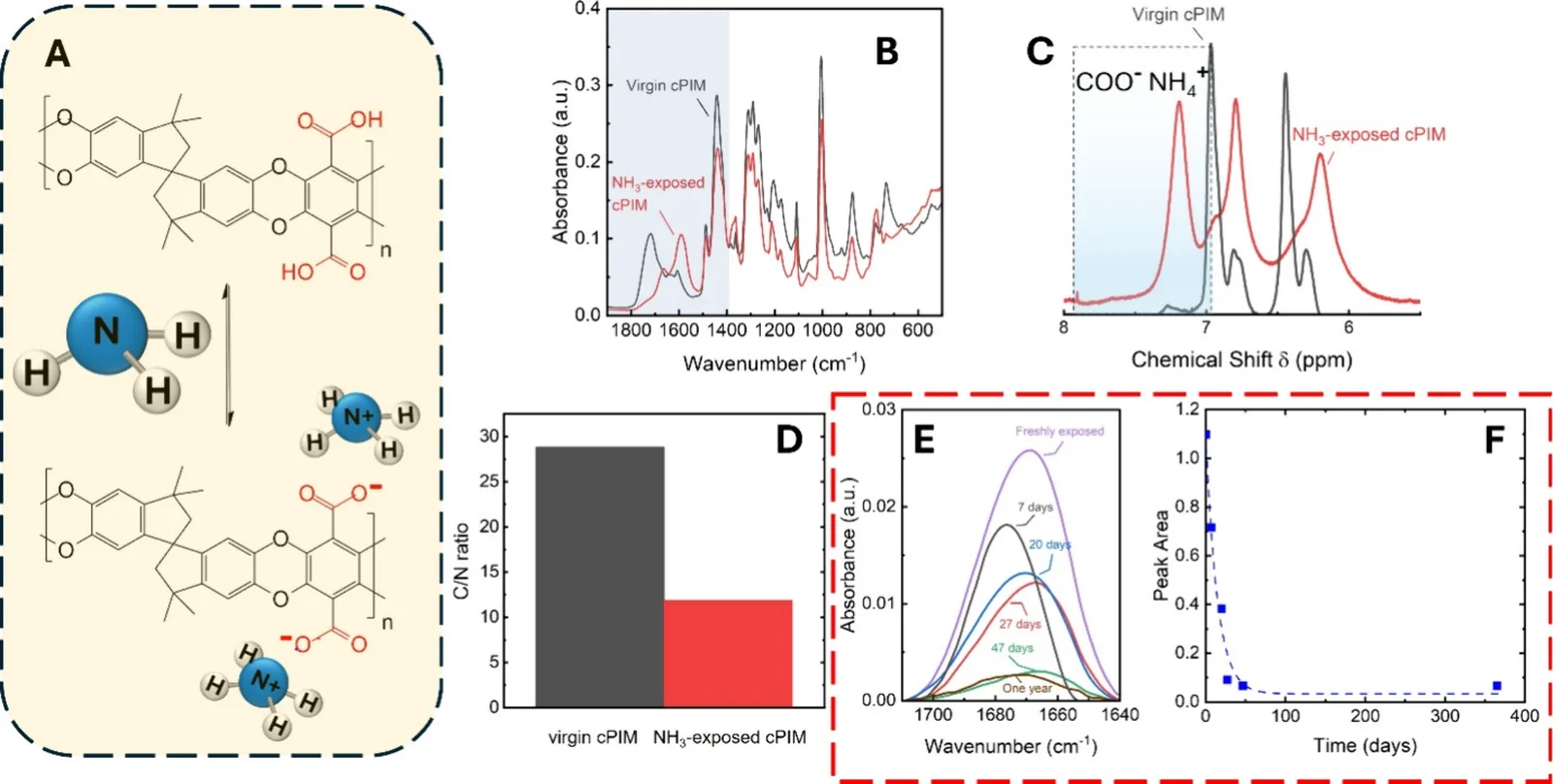
A) Schematic of cPIM reaction with ammonia, and formation of ammonium carboxylate. B) FTIR spectrum of cPIM before and after exposure to ammonia at 5 atm. C) ^1^H NMR spectrum of cPIM in deuterated DMSO before and after exposure to ammonia at 5 atm. D) Carbon/Nitrogen (C/N) ratio determined via combustion elemental analysis. E-F) Time-evolution of the ammonium carboxylate peak in cPIM upon ammonia removal.

The ^1^H NMR spectra of cPIM in deuterated DMSO, before and after exposure to ammonia, are shown in Figure 3C. The formation of ammonium carboxylate adducts in the ammonia-treated sample is confirmed by the appearance of a broad signal at 7.2 ppm.^12^

The carbon/nitrogen (*C/N*) ratio, determined via combustion elemental analysis, decreases by 55% in the ammonia-exposed cPIM relative to pristine cPIM (cf., Figure, 3D), confirming that carboxylic groups are converted into ammonium carboxylate upon exposure to ammonia. Based on the *C/N* ratio of virgin and ammonia-exposed cPIM and the experimental degree of hydrolysis, a simple carbon and nitrogen molar balance reveals that 100% of the carboxylate groups reacted with ammonia. This result aligns with the stoichiometric calculations derived from the ammonia sorption isotherms.

To understand the reversibility of the polymer modification upon ammonia removal, FTIR spectra of cPIM were collected for one year after the ammonia sorption test. In the meantime, the sample was stored in a Petri dish. As shown in Figure 3E, the gradual fading of the ammonium carboxylate peak (1670 cm^–1^) indicates that the polymer modification persists long-term despite its reversibility. During this decomposition process, cPIM fails to fully recover its original structure within the observed time frame. To better visualize this phenomenon, in Figure 3F the area of the peak (i.e., absorbance) is plotted as a function of time, showing an exponential decay.

Experimental details of structural characterization are provided in sections 4–9, Supporting Information.

The responsive behavior of the ammonium carboxylate-modified cPIM was subsequently evaluated by measuring pure-gas CO_2_ and H_2_ permeability at 35 °C. To put the results in broad perspective, CO_2_ and H_2_ permeability was measured in virgin cPIM (i.e., not pre-exposed to ammonia) and cPIM pre-equilibrated with ammonia at 5 atm. Direct mixed gas NH_3_/CO_2_ or NH_3_/H_2_ permeability measurements could not be run, as ammonia would damage the gas chromatograph used to analyze the permeate composition.

Contrary to the expectation that ammonia-induced plasticization would increase CO_2_ permeability, pre-exposure of cPIM to ammonia resulted in a nearly 10-fold reduction in CO_2_ permeability compared to the pristine polymer (cf., Figure 4A). Two hypotheses may explain this phenomenon: *i)* exposure of the ammonia-treated cPIM to CO_2_ triggers polymer ionic cross-linking via carbamate ions, thereby suppressing CO_2_ transport; *ii)* conversion of carboxyl groups to ammonium carboxylate renders the CO_2_–polymer interactions less favorable. Consequently, the observed decrease in CO_2_ permeability would mirror a suppression in either CO_2_ diffusivity (hypothesis *i*) or sorption (hypothesis *ii*) in cPIM, respectively.

**Figure 4:**
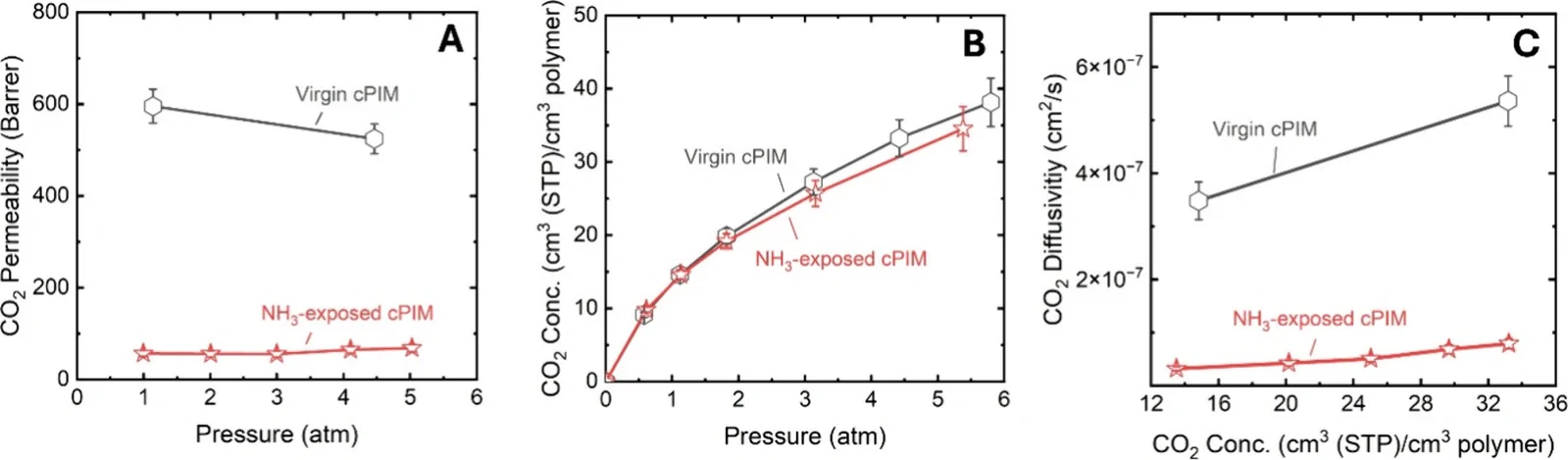
Carbon dioxide permeability (A) and sorption (B) in virgin and ammonia-treated cPIM at 35 °C as a function of pressure. C) Carbon dioxide diffusion coefficient in virgin and ammonia-treated cPIM at 35 °C as a function of carbon dioxide concentration in the polymer. Error bars were calculated using the error propagation method.^16^

To shed light on this aspect, CO_2_ permeability in virgin and ammonia-exposed cPIM was broken into its elementary sorption and diffusion contributions (cf., Figure 4B). At fixed pressure, CO_2_ uptake in the ammonia-treated cPIM is comparable to that measured in virgin cPIM, suggesting that the decrease in carbon dioxide permeability observed in the ammonia-treated sample is mostly due to a decrease in carbon dioxide diffusion coefficient (cf., Figure 4C). This result supports the hypothesis that CO_2_ reacts with the ammonium carboxylate units in NH_3_-pre-exposed cPIM to yield polymer ionic cross-linking, presumably via the formation of carbamate ions. Koros and co-workers observed similar effects in cross-linked polyimides, where cross-linking has negligible effects on gas sorption, while suppressing diffusivity.^22^

The most likely scenario is that ammonium carbamate ion pairs form a supramolecular ionic network with cPIM. Although deeper structural investigation is ongoing, gel-fraction measurements (cf. Figure 5A and section 7, Supporting Information) support this hypothesis. Specifically, while virgin cPIM is completely soluble in THF (i.e., the solvent used to cast the films), the ammonia and carbon dioxide treated sample exhibits a gel fraction near 76% in the same solvent. Both samples are soluble is d-DMSO which, exhibiting larger dielectric constant than THF, can dissociate ionic bridges.

**Figure 5:**
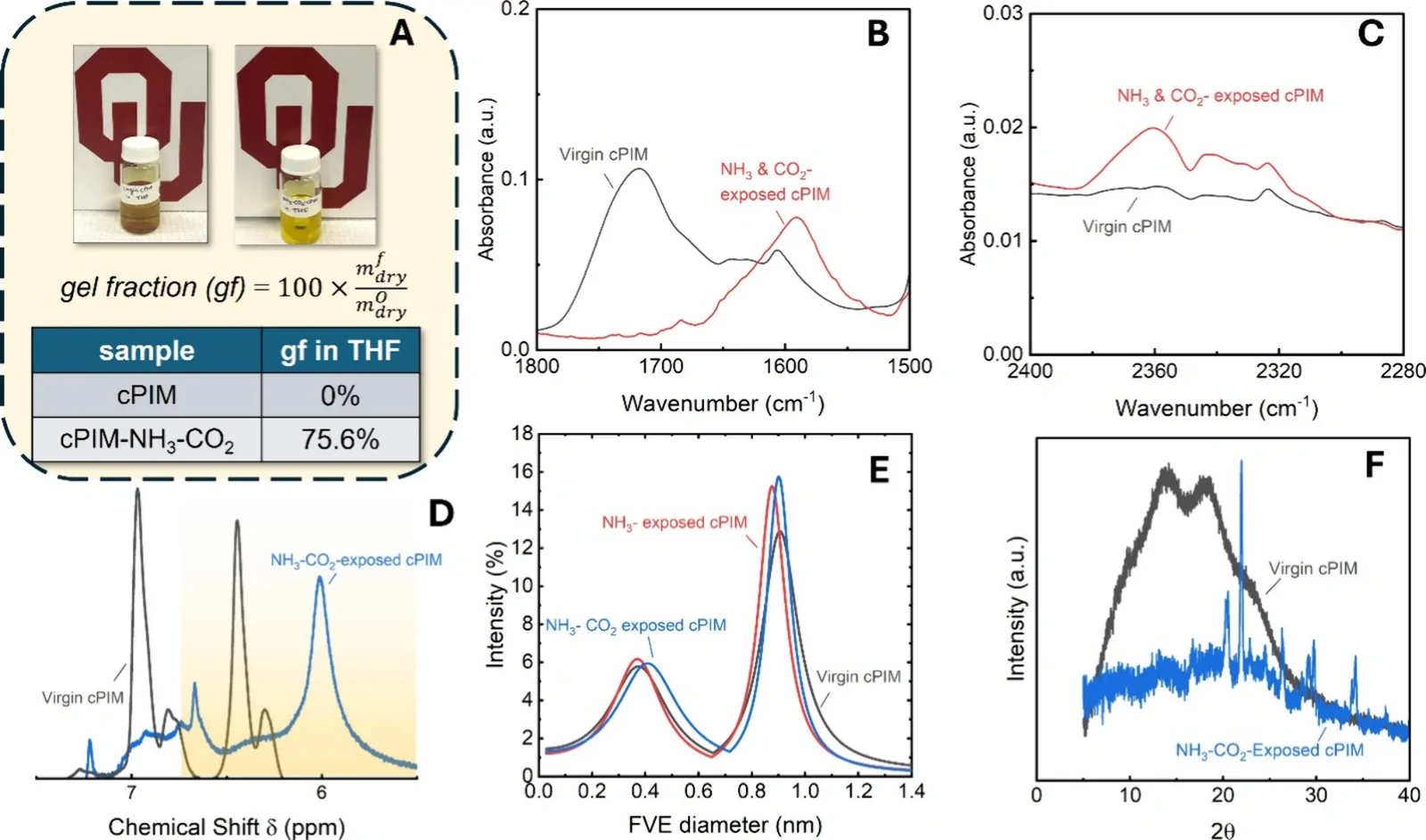
A) Gel fraction measurements in THF. B–C) FTIR and D) ^1^H NMR spectra collected on cPIM in its virgin state and after sequential exposure to NH_3_ at 5 atm and CO_2_ at 5 atm. d-DMSO was used as solvent for liquid state NMR measurements. E) PALS spectra of pristine cPIM, cPIM exposed to ammonia at 5 atm, and cPIM exposed sequentially to ammonia at 5 atm and carbon dioxide at 5 atm. F) XRD spectrum of cPIM in its virgin state and upon sequential exposure to NH_3_ and CO_2_.

FTIR spectra of cPIM exposed to ammonia and carbon dioxide are shown in Figure 5B,C. The peak associated with the asymmetric stretching vibration of the carbonyl group in the carbamate anion, which should show at 1550/1630 cm^–1^, overlapped with carboxylate asymmetric stretching, appears at 1590 cm^–1^. The N–H stretching vibration in the ammonium cation is expected to show near 2300 cm^–1^ when the cation is complexed with another species. Exposure of cPIM to ammonia and carbon dioxide yields a new peak at 2360 cm^–1^ (cf. Figure 5C), signaling possible ammonium carbamate generation.

The ^1^H NMR spectrum of ammonia and carbon dioxide-exposed cPIM after dissolution in d-DMSO is shown in Figure 5D. A weak ammonium ion peak is still detectable at 7.2 ppm, as in the cPIM sample after ammonia exposure. A new broad singlet appears at 6.0 ppm, which is compatible with the carbamate ion (NH_2_COO^–^) proton.^23^

PALS spectra were recorded for cPIM (cf. section 8, Supporting Information) in its pristine state, after exposure to 5 atm ammonia, and after subsequent exposure to 5 atm ammonia and 5 atm carbon dioxide (cf. Figure 5E). A bimodal free volume distribution is evident across the samples, indicating the coexistence of microporosity (7–20 Å) and of ultramicroporosity (<7Å).^24^ In the pristine cPIM sample, the average size of micropores and ultramicropores is 9 Å and 3.7 Å, respectively. Upon ammonia exposure, the average micropore size decreases to 8.7 Å while their abundance increases, which likely reflects the interplay between polymer swelling by ammonia and partial micropores occupation by bulky ammonium carboxylate. The latter aspect is confirmed by the decrease of the Langmuir sorption capacity observed during the second ammonia sorption run. The ultramicropore size and number remain unchanged.

Interestingly, exposing the ammonia-treated cPIM to carbon dioxide restores the micropore size to its original value (i.e., 9 Å) without changing their number relative to the ammonia-exposed sample. The size of ultramicropore increases from 3.7 to 4Å, although their abundance remains unchanged. This picture may reflect the elimination of ammonium carboxylate and the formation of a three-dimension ammonium carbamate-cPIM supramolecular network which, most likely, locks the cPIM structure preventing chain shrinkage and free volume collapse. Details of this process are under investigation.

Finally, Figure 5F shows the XRD pattern of virgin cPIM and cPIM after exposure to ammonia and carbon dioxide (cf., section 9, Supporting Information). While, according to previous reports^25^ virgin cPIM is amorphous, the XRD spectrum of the NH_3_/CO_2_-exposed cPIM exhibits some degree of structural order, likely associated with ionic crystallites. Peaks at 25–30° and 24° (2θ) indicate the presence of ammonium carbamate and ammonium carboxylate, respectively. Moreover, upon exposure to ammonia and carbon dioxide, cPIM turns opaque white, visually indicating precipitation of ionic crystalline domains. Importantly, this crystallite formation increases tortuosity along the CO_2_ diffusion pathway, which restricts gas transport, as shown in Figure 4.

In conclusion, while experimental data suggest carbamate-mediated ionic cross-linking, the precise mechanism driving cPIM cross-linking upon NH_3_ and CO_2_ exposure remains under investigation.

Since carbon dioxide transport in ammonia-exposed cPIM is accompanied by a chemical reaction, the *Damkohler number* (*Da*) of this process (i.e., the ratio between the reaction rate and the transport rate^26^) was estimated as $\frac{k_{R}C_{co_{2}}l^{2}}{D_{CO_{2}}}$ , where l is the membrane thickness, *C*_*CO*_2__ and *D*_*CO*_2__ are the CO_2_ concentration and diffusion coefficient in the ammonia-exposed cPIM, and *k*_*R*_ is the second-order reaction rate of CO_2_ with ammonium carboxylate. Since a realistic *k*_*R*_ value is not available, especially considering that the reaction occurs in a polymer, a parametric study was conducted.^27^ Varying *k*_*R*_ from 10^–1^ to 10^4^ L/(mol s) shifts *Da* from 10 to 10^6^. This result indicates a transport-limited process, as expected for a reactant diffusing through a network.

For comparison, hydrogen permeability in ammonia-exposed cPIM at 35 °C and 5 atm is 35-fold higher than in pristine cPIM (17,400 vs 514 Barrer, cf., Figure S1, Supporting Information). Unlike carbon dioxide, hydrogen cannot induce polymer cross-linking, allowing ammonia-induced plasticization to enhance hydrogen permeability relative to pristine cPIM.

In summary, we elucidated how cPIM adapts structurally and functionally to environmental changes, demonstrating that this response persists poststimulus. Given the practical relevance of CO_2_/NH_3_ separation, this study underscores that evaluating pure-gas transport in cPIM without accounting for coupling effects yields misleading conclusions.

## Supplementary Material



## Notes

Synthesis of carboxylate polymer of intrinsic microporosity (cPIM) and membrane formation; Pure-gas sorption measurements; Pure-gas permeability measurements; Elemental analysis measurements; Liquid state NMR measurements; FTIR measurements; Gel fraction measurements; Positron Annihilation Lifetime Spectroscopy (PALS); XRD measurements; H_2_ permeability data (PDF)
