# Supplementary material for: Penetrant-Triggered Responsiveness in Carboxylate Polymer of Intrinsic Microporosity
Source: ACS Macro Lett. 2026 Oct 8;15(10):1610–6. doi: 10.1021/acsmacrolett.6c00475 (PMC13689930; doi:10.1021/acsmacrolett.6c00475)
Supplement: Supplementary file 1 [file mz6c00475_si_001.pdf]

Supporting Information

Penetrant-triggered responsiveness in carboxylate polymer of intrinsic microporosity

Maria S. Tavera-Villamizar[1], Durga Acharya[2], Cara M. Doherty[2],

Anita J. Hill[2] and Michele Galizia[1,3]*

[1] School of Sustainable Chemical, Biological and Materials Engineering, University of Oklahoma, 100E. Boyd Street, Norman, 73019, OK, USA

[2] CSIRO Manufacturing, Research Way 3168, Clayton, Australia

[3] Materials Science and Engineering Program, The University of Oklahoma, 660 Parrington Oval, Norman, 73019, OK, USA

*Corresponding Author: *mgalizia@ou.edu*

Table of Contents:

1. Synthesis of carboxylate polymer of intrinsic microporosity (cPIM) and membrane formation.

2. Pure-gas sorption measurements.

3. Pure-gas permeability measurements.

4. Elemental analysis measurements.

5. Liquid state NMR measurements.

6. FTIR measurements.

7. Gel fraction measurements.

8. Positron Annihilation Lifetime Spectroscopy (PALS).

9. XRD measurements.

10. H_2 permeability data.

1. Synthesis of carboxylate polymer of intrinsic microporosity (cPIM) and membrane formation

Details of cPIM synthesis are provided in previous studies[1]. For the sake of brevity, they are summarized in the following.

Chloroform (ACS, > 99.8%), N,N-Dimethylacetamide (DMAC, HPLC, > 99.5%), and 5,5',6,6'-Tetrahydroxy-3,3,3',3'-tetramethyl-1,1'-spirobisindane (TTSBI, 97%), were purchased from Alfa Aesar. Methanol (HPLC, > 99.9%) and toluene (HPLC, 99.8%) were purchased from Fischer chemical. Tetrafluoroterephthalonitrile (TFTPN, 98%) was purchased from TCI Chemicals. Potassium carbonate (K_2CO_3) was purchased from Sigma Aldrich. Tetrahydrofuran (THF, HPLC, inhibitor-free) was purchased from Macron Fine Chemical. ACS grade sulfuric acid was purchased from EMD, and glacial acetic acid from Research Products International.

TFTPN and TTSBI were dried at 30 °C and 110 °C, respectively, in a vacuum oven overnight before use, without any further purification. The amounts of TFTPN and TTSBI used in the synthesis of PIM-1 were adjusted for the listed purity. Potassium carbonate, needed to neutralize the hydrofluoric acid formed during the synthesis of PIM-1, was ground in a mortar and pestle, sieved through a 220 mesh (0.065 mm) Titan brand stainless-steel sieve, and dried at 110 °C in a vacuum oven overnight before use.

PIM-1 was synthesized via high-temperature polycondensation. Glassware was dried at 130°C in an oven overnight before use. A 250 ml 3-neck flask was equipped with a mechanical stirrer, purge gas inlets/outlets, reflux and addition funnel. 13.44 mmol of TFTPN, 13.43 mmol of TTSBI, and 27 ml of DMAc were added, then a 5 ml/second dry nitrogen purge was delivered to the flask. The monomers were stirred until they fully dissolved to form an orange-brown solution. The stir rate was then set to 200 rpm, and 40.52 mmol of the pre-dried, ground, and sieved K_2CO_3 was added. Following this step, 14 ml of toluene was added in a single shot using the addition funnel, and the flask was submerged in a pre-heated oil bath at 160°C. A viscous yellow solution formed within 5 minutes. Following this step, 9 ml of toluene was added to dilute the solution and reduce the viscosity. After 30 minutes of stirring, additional 18 ml of toluene was added, and the flask was removed from the oil bath. The reaction mixture was precipitated into 500 ml of methanol to yield bright yellow fibers, which were solvent exchanged with water to remove residual salts from the reaction. Specifically, the fibers were soaked in hot water (95°C) for 1 hour. The water in this bath was decanted off and changed, repeating the exchange process three times. The fibers were dried at 130°C in a vacuum oven overnight. The resulting PIM-1 was then dissolved in 250 ml of

chloroform and precipitated into a 1:1 (v:v) solution of acetone:THF to extract low-molecular weight residues. The solvents were decanted, and the resulting fibers were then soaked in a water bath at 95°C. The exchange process was repeated three additional times to further remove any residual salts. Finally, the fibers were dried at 130°C in a vacuum oven overnight to yield the finished product (86.4% final yield).

Following these steps, carboxylate PIM (cPIM) was prepared via the acid hydrolysis method, as discussed in a previous study[1]. Specifically, 0.8 g of PIM-1 was combined with 48 mL of deionized (DI) water, 16 mL of glacial acetic acid, and 48 mL of sulfuric acid in a 250 mL single neck round-bottom flask equipped with a water-cooled condenser and magnetic stir bar. The reaction was heated by submerging the flask in an oil bath at 150°C under stirring at 200 rpm. After 48h, the reaction mixture was decanted into 500 mL of water. The resulting solution was filtered to recover a brown-colored product. To retain the protonated form of the acid groups, the product was refluxed in 200 mL of DI water with 3-4 drops of sulfuric acid for 24h. Finally, the final product was recovered by filtration and dried in a vacuum oven at 110°C overnight. The degree of carboxylation, determined via elemental analysis, was about 56% .

The cPIM structure was confirmed via FTIR and NMR, as shown in ref. 1, and in Fig. 3, main manuscript.

Free-standing, dense cPIM films, 30-40 μm thick, were fabricated via the solution casting method from a 3wt.% solution in THF, in a leveled Teflon *Petri dish*. Membranes were cast in a sealed glove bag, which had been previously purged with high purity N_2. Following this step, TGA was run on the membranes to verify complete solvent removal.

2. Pure-gas sorption measurements.

The pure-gas sorption apparatus consists of two chambers of known volume connected via a valve, which are identified as the “sampling” and “charge” chambers. Both chambers are equipped with PX-409-USHB series Omega pressure transducers (0-750 psi, ± 0.08% BSL accuracy). The polymer sample is hosted in the sampling chamber. Both chambers are then evacuated overnight using a rotary vacuum pump (Welch DuoSeal 1402).

The desired gas or vapor (i.e., NH_3 or CO_2) is then introduced to the charge chamber and allowed to reach thermal equilibrium before quickly releasing it into the sampling chamber, upon opening the valve between the two chambers for a few seconds. The valve separating the two chambers is

then closed, and the pressure in each chamber is allowed to reach equilibrium. Pressure decay in the sampling chamber is observed due to gas partitioning between the external gas phase and the polymer sample. Upon reaching sorption equilibrium, the process is repeated stepwise, without pulling vacuum. The overall sorbed gas concentration in the polymer sample is calculated using the initial charge pressure and equilibrium pressures for each chamber, at each step, as follows:

$$C_{pol,j} = \frac{\rho_{pol}}{g_{pol,dry}}\left(\sum_{i=0}^{j}\left(\frac{1}{\tilde{V}_{C0,i}} - \frac{1}{\tilde{V}_{CF,i}}\right) - \frac{V_S}{\tilde{V}_{SF,j}}\right) \qquad (Eq. S1)$$

where ρ_{pol} is the polymer density (assumed constant), $g_{pol,dry}$ is the dry polymer mass, j is the current pressure step, V_s is the sample chamber volume, V_C is the charge chamber volume, and $\tilde{V}_{C0}$, $\tilde{V}_{CF}$, $\tilde{V}_{SF}$, are the gas molar volumes (calculated using the Peng-Robinson equation of state) for the initial charge state (prior to valve opening), final charge state, and final sample state, respectively. Experimental uncertainties were calculated according to the method reported by Box et al.[2].

When pulling vacuum throughout the system, residual ammonia was neutralized in a H_2SO_4 aqueous solution directly connected to the vacuum pump exhaust. To immediately detect any possible NH_3 leakage, an ammonia detector was placed next to the sorption apparatus. The maximum pressure reached during ammonia sorption and transport measurements was 5 atm, which is well below the saturation pressure at 35°C (i.e., 13.4 atm). Carbon dioxide sorption in virgin and ammonia-treated cPIM was measured at 35°C using the same methods described for ammonia.

To measure CO_2 sorption in the ammonia-pretreated sample, the following procedure was used. First, cPIM was exposed to ammonia at 5 atm and 35°C, directly in the sorption apparatus. Upon reaching sorption equilibrium (~ 9 hours), vacuum was pulled throughout the system for 24 hours at 35°C. Following this pre-treatment, CO_2 was fed to the system to measure sorption in a stepwise manner, as explained above for pristine cPIM.

3. Pure-gas permeability measurements.

A round membrane sample was hosted in a 47 mm HP filter holder. Prior to measurements, vacuum was pulled on the system overnight using a rotary vacuum pump (Welch DuoSeal 1402) to remove any sorbed species from the membrane. Measurements were performed by charging the desired gas pressure to the upstream side of the membrane. Upstream pressure was monitored using a PX-

409-USHB series Omega pressure transducer (0-1000 psi). Then, gas was allowed to permeate through the membrane to the downstream volume, where the increase in pressure with time was measured using an MKS Baratron capacitance manometer (0-10 Torr). The permeability (P, in units of Barrers) was calculated as follows:

$$P = \frac{\ell V_{down}}{\Delta p A R T}\left[\left(\frac{dp}{dt}\right)_{down} - \left(\frac{dp}{dt}\right)_{leak}\right] \qquad (Eq. S2)$$

where ℓ is the membrane thickness, A is the membrane area available to gas transport, R is the ideal gas constant, T is the absolute temperature, Δp is the average transmembrane pressure difference, $\frac{dp}{dt}_{down}$ is the asymptotic rate of pressure increase in the downstream volume, $\frac{dp}{dt}_{leak}$ is the rate of pressure increase in the downstream volume whilst the charge pressure is 0 (i.e., leaking rate, which was always at least 100 times lower than the gas permeation rate), and V_{down} is the downstream volume. Membrane thickness was determined by the average of 10 measurements made using a Mitutoyo micrometer with $\pm$ 1 µm resolution.

Before the permeation test, the membranes were glued with epoxy resin on a brass custom-made ring, then scanned at a resolution of 600 dpi. The passage area was thus calculated as the average of 9 measurements collected from 3 measurements on 3 different scans of the brass coupon at different rotations, to average out any ambiguities due to shadows and color gradients.

Ammonia and carbon dioxide permeation in cPIM was measured at 35°C using the method described above. To measure pure CO_2 and H_2 permeability in cPIM pre-exposed to NH_3, the following protocol was adopted. First, cPIM was exposed to ammonia at 5 atm and 35°C, directly in the permeation apparatus. After ~ 9 hours, vacuum was pulled throughout the system for 24 hours at 35°C. Following this pre-treatment, pure CO_2 or H_2 was fed to the system to measure permeability in NH_3 pre-conditioned cPIM.

Experimental uncertainties were calculated according to the method reported by Box et al.[2].

4. Elemental analysis measurements.

Elemental analysis was conducted first to assess the degree of cPIM hydrolysis. Samples were analyzed using a Vario EL Elemental CNS Analyzer (Elementar). Before cPIM analysis, the instrument was calibrated by injecting three separate samples of sulfanilamide. The degree of

hydrolysis (DH) was determined by calculating the fraction of nitriles converted to carboxylic groups, as follows:

$$DH\ (\%) = \frac{(N/C)_{PIM-1} - (N/C)_{cPIM}}{(N/C)_{PIM-1}} \qquad (Eq.\ S3)$$

where N/C is the nitrogen to carbon molar ratio. Elemental analysis was also used to calculate the N/C ratio in the cPIM sample exposed to ammonia at 5 atm and 35°C, using the same procedure described above.

5. Liquid state NMR measurements.

NMR spectra were acquired for the virgin cPIM, cPIM exposed to ammonia at 5 atm and 35°C, and cPIM exposed to ammonia and carbon dioxide, both at 5 atm and 35°C, to study how the external chemical environment changes the polymer chemical structure. All liquid-state NMR measurements were carried out on a 500MHz JEOL spectrometer at 292.25 K using deuterated DMSO (DMSO-d6) as the solvent. The ^{1}H NMR spectra were recorded as the average of 32 scans with an acquisition time of 1.9987 s, a relaxation delay of 1.0 s, and a pulse width of 3.9505 μs.

Virgin cPIM: ^{1}H NMR (500 MHz, DMSO-d_6) δ.

NH_3-exposed cPIM: ^{1}H NMR (500 MHz, DMSO-d_6) δ 7.2 (br s, NH_4^+).

NH_3-CO_2-exposed cPIM: ^{1}H NMR (500 MHz, DMSO-d_6) δ 7.3 (NH_4^+), 5.8 (br s, NH_2COO^-).

6. FTIR measurements

FTIR-ATR spectra of virgin cPIM, cPIM exposed to ammonia at 5 atm and 35°C, and cPIM sequentially exposed to ammonia to 5 atm and carbon dioxide at 5 atm were collected using a Thermo Scientific Nicolet iS50R Fourier Transform Infrared Spectrometer with a diamond crystal in the absorbance mode. Spectra were recorded by averaging 64 scans at a resolution of 4.0 cm^{-1}. Polymer film samples were placed directly on the ATR crystal interface, and spectral data were processed using the OMNIC software. For the ammonia-treated sample, measurements were run for 1 year, to test the reversibility of the chemical modification experienced by the polymer. Between measurements, the sample was stored in a *Petri dish* under normal ambient conditions.

7. Gel fraction measurements.

Gel fraction of cPIM membranes were measured to confirm ionic crosslinking resulting from sequential exposure to NH_3 at 5 atm and CO_2 at 5 atm. Freshly fabricated membranes were first dried at 110 °C in a vacuum oven for over 4 hours to remove residual moisture and solvent. The dried membranes were then placed in a pressure chamber and exposed subsequently to NH_3 gas at 5 atm, evacuated, and subsequently exposed to CO_2 gas at 5 atm, with each exposure step held for 24 hours to ensure equilibrium was reached. After exposure, the membranes were weighed to obtain the initial mass, m_0. The membranes were then soaked in liquid THF for 24 hours, dried at 110 °C under vacuum overnight, and finally weighed again to obtain the final mass, m_f. The gel fraction was finally calculated according to Eq. S4:

$$gel\,fraction\,(\%) = \frac{m_f}{m_0} \times 100 \qquad (Eq.\,S4)$$

The rationale for selecting THF is that this solvent can fully solubilize virgin cPIM. In fact, it was used to cast the films used throughout this study.

8. Positron Annihilation Lifetime Spectroscopy (PALS).

PALS measurements were performed using an automated EG&G Ortec fast−fast coincidence spectrometer under vacuum (10^{-6} Torr) and at room temperature to measure the lifetimes (τ) and relative number of pores (Intensity, I). Three samples were tested: pristine cPIM, NH_3-treated

cPIM (at 5 atm), and finally NH_3- and CO_2-treated cPIM (both at 5 atm, as described above). A minimum of 4.5×10^6 integrated counts were collected for each spectrum and at least five spectra were collected per sample. The data were then fitted using the LT-9 software with a source correction for the Na-22 source sealed in Mylar (1.478 ns, 3.24%). Ortho-positronium (oPs) lifetime components were fitted and related to radii of free volume elements (R) which are assumed spherical, through the Tao-Eldrup equation:

$$\tau = \frac{1}{2}\left(1 - \frac{R}{R+\Delta r} + \frac{1}{2\pi} \sin\left[\frac{2\pi R}{R+\Delta r}\right]\right)^{-1} \qquad (Eq. S5)$$

where *Δr* is the electron layer thickness (1.66 Å) which is determined empirically. The cavity size distribution was calculated using the *PAScual* software[3].

9. XRD measurements.

X-ray diffraction measurements were collected on a Rigaku Smartlab XE diffractometer equipped with a rotating anode (Cu Kα radiation, λ= 1.54059 Å) operated at 45kV and 200 mA with a fine focus of 0.4 mm x 8 mm. Measurements were performed in a Bragg-Brentano reflection geometry with a goniometer radius of 300 mm. The incident beam was conditioned with cross-beam optics (CBO-A) and passed through a divergence slit of 1/2° and 10 mm length-limiting slit. Diffraction patterns were recorded using a HyPix-3000 semiconductor detector operating in 1D (line) integration mode. Polymer film samples were mounted flat onto a zero-background silicon holder and scanned over a 2θ range of 5.0° to 80.0° with a step size of 0.01° and a scan speed of 10.0°/min.

10. Hydrogen permeability data

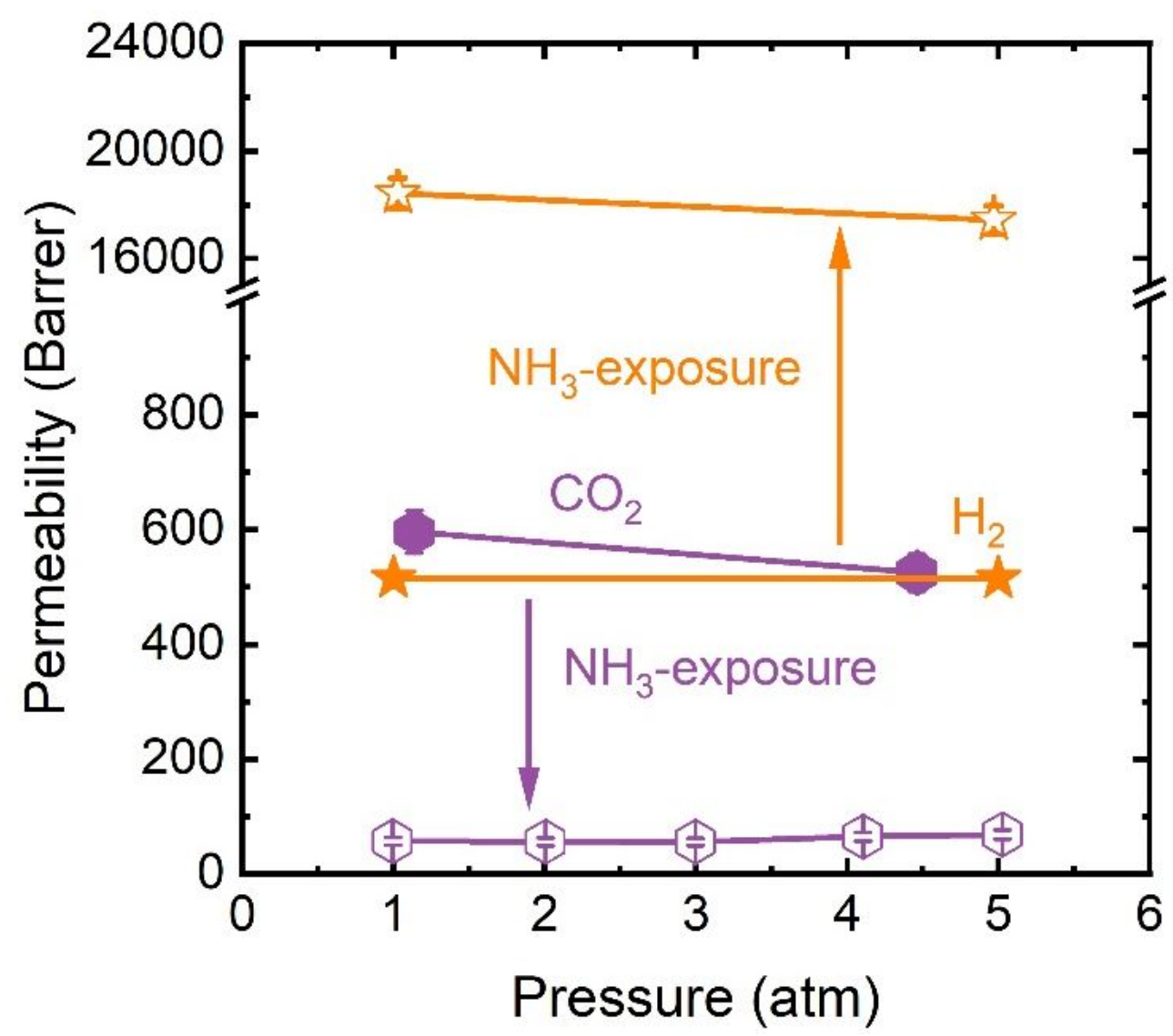

Figure S1. *Effect of ammonia exposure on H_2 permeability in cPIM. For the sake of comparison, data for CO_2 are also shown.*

References

1. Condes, L. C.; Landry, A. E.; Webb, M. T.; Box, W. J.; Gali, A.; Galizia, M., Inter-crosslinked spirocyclic mixed matrix membranes exhibiting enhanced gas permeability, selectivity, and plasticization resistance. *Journal of Membrane Science* **2024,** *709*, 123081.

2. Box, W. J.; Webb, M. T.; Galizia, M., Evaluating the experimental uncertainty in gas and vapor sorption/adsorption measurements: fundamental considerations and experimental design implications. *Industrial & Engineering Chemistry Research* **2022,** *61* (27), 9856-9868.

3. Condes, L. C.; Webb, M. T.; Le, T. B.; Box, W. J.; Doherty, C.M.; Gali, A.; Garrido, L.; Deng, J.; Matesanz-Nino, L.; Lozano, A.E.; Alvarez, C.; Buongiorno Nardelli, M.; Striolo, A.; Hill, A.J.; Galizia, M., Elucidating the Molecular Mechanisms by which Porous Polymer Networks Affect Structure, Aging Propensity, and Selectivity of Microporous Glassy Polymer Membranes using a Multiscale Approach. *ACS Applied Materials & Interfaces* **2024**, 16 (40), 53843-53854.
